# Deciphering the phenol degradation metabolic pathway in *Scedosporium apiospermum* HDO1

**DOI:** 10.1128/aem.01038-25

**Published:** 2025-07-10

**Authors:** Laura L. Diaz Ortiz, David Botero-Rozo, Natalia Vargas, Sandra Ortiz, Silvia Restrepo, Martha J. Vives

**Affiliations:** 1 Centro de Investigaciones Microbiológicas, Universidad de los Andes 27991, Bogotá, Colombia; 2 Laboratorio de Micología y Fitopatología Uniandes, Universidad de los Andes 27991, Bogotá, Colombia; 3 Departamento de Química, Universidad de los Andes 27991, Bogotá, Colombia; Shanghai Jiao Tong University, Shanghai, China

**Keywords:** phenol, fungal biodegradation, differentially expressed genes, catechol, hydroquinone

## Abstract

The filamentous fungus *Scedosporium apiospermum* is a microorganism capable of phenol degradation. Phenol is a petroleum-derived pollutant and a compound widely used in several industries. As a result of its widespread use, phenol is commonly discarded and accumulated in soils and water bodies. In this study, overexpressed and repressed genes that produce enzymes involved in phenol metabolism were identified in *S. apiospermum* HDO1 when the fungus grows in the presence of phenol. The fungus was grown with either glucose (control) or phenol as the sole carbon source to achieve this. RNA from the mycelium was extracted and sequenced using the Illumina Hiseq-4000 platform, with paired-end libraries. Eighteen genes coding for enzymes related to catechol ortho-cleavage, catechol meta-cleavage, and hydroquinone pathways were annotated from the assembled transcriptome. In the differential gene expression analysis, 11 genes coding for phenol 2-monooxygenase, catechol 1,2-dioxygenase, 3-oxoadipate enol lactonase, hydroxyquinol 1,2-dioxygenase, and aldehyde dehydrogenase were overexpressed. In contrast, one gene coding for protocatechuate 3,4-dioxygenase was repressed. We show for the first time that phenol degradation in *S. apiospermum* occurs through one of the catechol routes, the catechol-ortho ring cleavage pathway, and through the hydroquinone A pathway. These findings are important because they improve the understanding of how eukaryotic microorganisms with the potential for bioremediation degrade organic pollutants such as phenol.

**IMPORTANCE:**

In recent years, bioremediation has emerged as one of the solutions to eliminate pollutants from the environment. *Scedosporium apiospermum* is one of the fungi capable of tolerating and degrading common pollutants such as phenol. This ability is of great interest as it highlights its potential for use, but also as an important eukaryotic model in contaminant metabolism. *S. apiospermum* has been widely studied for its clinical significance, but little is yet known about its role in natural environments and its capacity for removing organic pollutants. Using previously published biochemical data together with our differential gene expression results, we validated and completed the proposed phenol metabolic pathways.

## INTRODUCTION

Phenol is an aromatic hydrocarbon that pollutes natural water bodies and is mostly generated as a waste product of industrial activities (1). The accumulation of phenol in water bodies for prolonged periods represents an important risk for both the environment and human health; the Environmental Protection Agency of the United States and the European Union have cataloged phenolic compounds as pollutants of global priority importance due to their toxicity and short- and long-term effects on animals and humans (1, 2). They can cause poisoning in humans through skin absorption, inhalation, and ingestion (2, 3).

The use of microorganisms is key among the bioremediation strategies focused on removing contaminants. The degradation of hydrocarbons such as phenol by fungi has been widely explored (4–6). *Scedosporium apiospermum* (family Microascaceae, class Sordariomycetes) is an opportunistic pathogen (7) and saprotrophic filamentous fungus (8) which grows commonly in soil, wastewater, and other hydrocarbon-polluted environments (8, 9). Several studies have shown the phenol degradation capabilities of *S. apiospermum* (8, 10, 11) and its potential to be used as part of bioremediation strategies in polluted environments (10, 11).

In 1998, Claussen and Schmidt described two possible phenol degradation pathways that occur simultaneously in *S. apiospermum* through enzymatic assays and metabolite and substrate identification using high-performance liquid chromatography (HPLC) (8). These were the catechol ortho-cleavage and the hydroquinone pathways (8). Both pathways start with the addition of one atom of molecular oxygen (O_2_) to the phenol ring by the phenol 2-monooxygenase enzyme. Phenol 2-monooxygenase, also known as phenol hydroxylase, belongs to flavoprotein aromatic hydroxylases enzymes (12, 13). This enzyme uses phenol, NADPH, and O_2_ as substrates. In the reaction, NADPH is oxidized to NADP^+^, an oxygen atom and a hydrogen atom are introduced into the phenol aromatic ring producing catechol, and the other oxygen atom produces water (12, 13). Depending on the position where the oxygen and hydrogen atoms are inserted into the aromatic ring, this reaction can produce catechol or hydroquinone (5). In the following reactions, the aromatic ring is cleaved, producing intermediates that will be transformed into molecules that enter the tri-carboxylic acid cycle (14).

Vandeputte et al. (15) assembled and annotated the genome of *S. apiospermum* IHEM 1462, obtained from a clinical sample in 2014 (15). They annotated 10,919 coding sequences (CDSs) and assigned a function to 8,818 of them (15). This annotation includes the genes coding for phenol 2-monooxygenase (SAPIO_CDS8718), catechol 1,2-dioxygenase (SAPIO_CDS5831—part of the catechol pathway), maleylacetate reductase (SAPIO_CDS9497—part of the hydroquinone pathway), and hydroxyquinol 1,2-dioxygenase (SAPIO_CDS8719—part of the hydroquinone pathway) (10, 15).

Later, Morales et al. (16) sequenced and assembled the genome of the environmental strain *S. apiospermum* HDO1 (16). This strain was obtained from a laboratory assay of crude oil biodegradation; 11,195 genes were identified as CDSs, and several genes that might be involved in phenol degradation were annotated. Among these, four were genes coding for the phenol 2-monooxygenase enzyme, and two were genes encoding for the catechol 1,2-dioxygenase (16).

Although the evidence for the phenol degradation mechanism in *S. apiospermum* is the detection of some metabolites described in the catechol and hydroquinone pathways (8), and some of the genes described for these pathways have been annotated in the assembled IHEM 1462 and HDO1 genomes (15, 16); it remains unclear whether the genes coding for enzymes of these pathways are involved in the phenol metabolism, or if other genes belonging to different pathways might be involved in the process. For example, the Cytochrome P450 (CYP450) family, which participates in catabolic reactions of organic compounds in white rot fungi (17). For this reason, the objective of this work was to identify the phenol degradation pathways in *S. apiospermum* HDO1 through transcriptomics (RNAseq) and differential gene expression analysis. The identification of overexpressed genes related to phenol metabolism will improve our understanding of the strategies used by *S. apiospermum* to grow in hydrocarbon-polluted environments and to metabolize such pollutants.

## MATERIALS AND METHODS

### Experimental design

**Fig 1:**
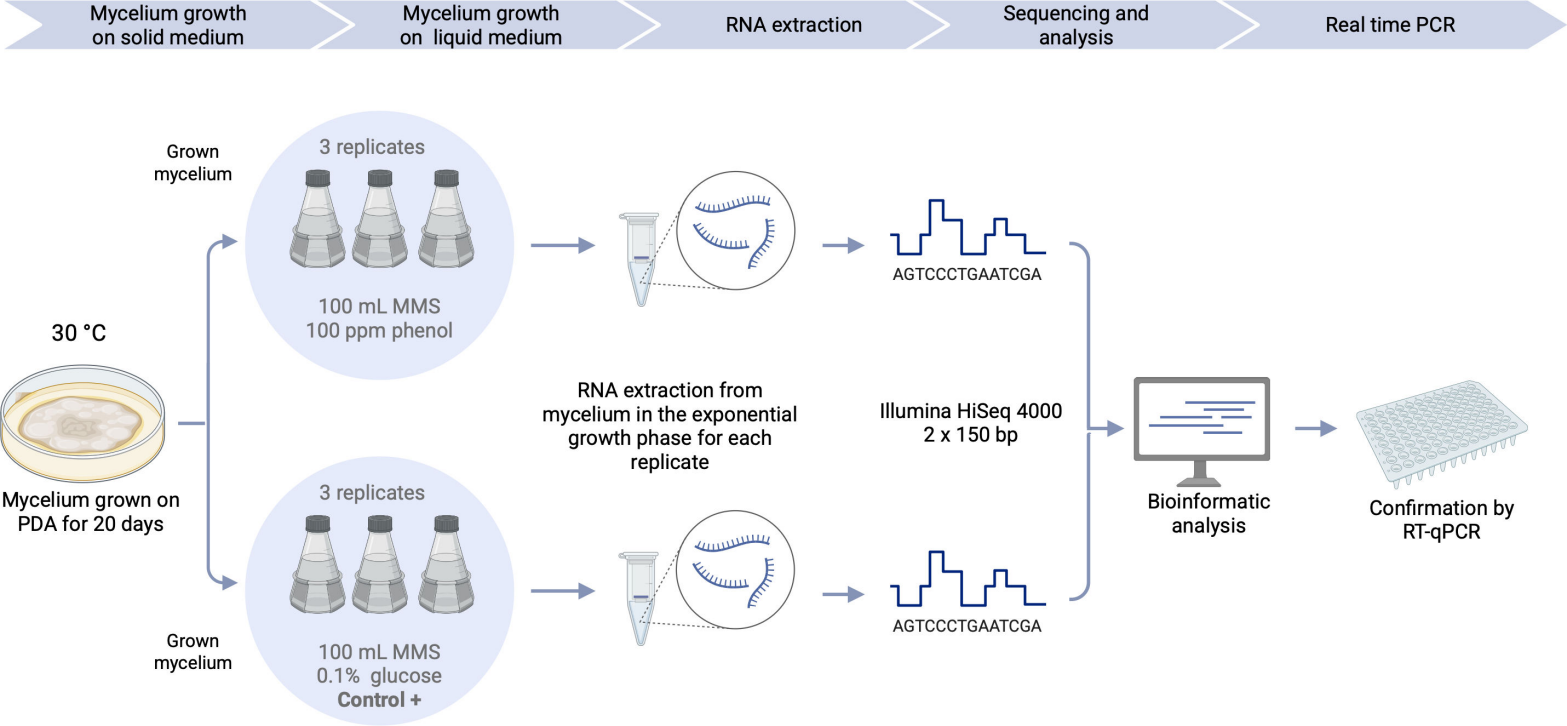
Experimental design of the transcriptomic analysis to identify the phenol-degrading genes in *Scedosporium apiospermum* HDO1. The fungus was grown on liquid MMS medium under two conditions: with phenol (experimental condition) and with glucose (control condition). The mycelium was grown at 30°C at 100 rpm in both conditions. Three biological replicates were prepared for each condition. Fungal biomass was collected once the mycelium reached the exponential growth phase, and RNA extraction was performed. Then RNAseq sequencing, bioinformatic analysis, and confirmation of RNA-Seq results via RT-QPCR were conducted. Created in https://BioRender.com.

*S. apiospermum* environmental strain HDO1 was grown in two different conditions: cultures supplemented with phenol at 100 ppm (experimental condition) or glucose at 0.1% (control) as the sole carbon source. Three biological replicates were made for each condition (F1, F2, F3 for phenol, and G1, G2, G3 for glucose) (Fig. 1).

### *S. apiospermum* HDO1 growth

The strain used in this study was previously identified as *S. apiospermum* HDO1 through sequencing of the ITS region and characterization of its morphological features as described by Morales et al. (16). Growth assays were performed to determine the growth curve of the fungus and to find the exponential growth phase for subsequent experiments. The HDO1 strain was maintained aerobically at 30°C in potato dextrose agar (PDA) (OXOID Ltd, Hampshire, UK) plates for approximately 20 days. After the mycelium in PDA reached a diameter of 7 cm, the samples were grown in minimal salt medium (MMS). MMS composition is 2 g/L NH_4_NO_3_, 0.5 g/L MgSO_4_, 0.6 g/L HK_2_PO_4_, 0.5 g/L H_2_KPO4, 0.1 g/L CaCl_2_, and 1 mL/L trace element solution (CuSO_4_·5H_2_O, MnCl_2_·H_2_O, H_3_BO_3_, NaMoO_4_, FeCl_3_, and ZnCl_2_), supplemented with the carbon source either 0.1% glucose or 100 ppm phenol. The liquid cultures were incubated at 30°C and 100 rpm (Fig. 1). The mycelium was collected daily for 8 days by destructive sampling. Daily, three replicates for each condition were collected (100 mL each), and the fungal biomass was weighed.

The mycelium was harvested by sterile vacuum filtration to determine the fungal biomass of each culture. The filtered mycelia were stored in Falcon tubes at −80°C until their processing. The stored mycelia were then lyophilized and weighed to obtain their dry weight. The exponential growth phase was determined using the dry weight in grams for each day. After determining the exponential growth phase of the HDO1 strain on glucose and phenol, new cultures were carried out, and biomass was collected at the exponential growth phase for RNA extraction and transcriptome sequencing.

### RNA extraction and sequencing

RNA extraction and Illumina sequencing were performed by GENEWIZ, LLC. (South Plainfield, USA). RNA was extracted from fresh-frozen mycelial tissue (10 mg per sample), and a strand-specific RNA library with poly(A) selection was prepared. Ribo-Zero rRNA removal kit was used. The sequencing was done using Illumina HiSeq 4000, 2 × 150 bp configuration, dual index, per lane.

### Transcriptome assembly and read abundance

The bioinformatic pipeline used in this work can be found in Fig. S1. The quality of the raw reads was assessed using FastQC v0.11.7 (https://www.bioinformatics.babraham.ac.uk/projects/fastqc/) before and after the removal of adapters and low-quality sequences. Reads of low quality and the adapter were trimmed by Trimmomatic v0.32 (18). The remaining reads were mapped against 28S, 18S, 16S, and 23S databases from the SILVA rRNA database project, and the ribosomal sequences were removed using SortMeRNA v2.1 (19). Filtered reads were *de novo* assembled using Trinity v2.8.4 implemented in Singularity (20) using the default *k-mer* size of 25. Transcriptome assembly statistics were examined for the newly formed transcriptome: the total number of contigs assembled, percentage of GC content, average contig length, and *N*_50_ number. Identification of the open reading frames (ORFs) from the assembled transcripts was achieved using TransDecoder v3.0.2 (https://TransDecoder.github.io/). The reads were mapped to the identified ORFs using Bowtie2 (21), and transcript abundance per sample was calculated with RSEM (20, 22).

### Functional annotation

Genes of interest were identified by searching those coding for enzymes involved in the phenol degradation pathway described by Claussen and Schmidt (8) and completed using enzymes and metabolic paths of the KEGG Benzoate degradation pathway (23) (https://www.kegg.jp/pathway/map00362). Functional annotation was done using PANNZER2 (24) and eggNOG-mapper v2 (25). Pannzer is an annotation web server that uses SANSparallel to do an interactive homology search against Uniprot (26). PANNZER2 applies the positive predictive value (PPV), a normalized prediction score between 0 and 1, to estimate the reliability of the predicted annotation (24). The annotation of eggNOG-mapper v2 was assessed with the Diamond *e* value (27). A lower *e* value means that the annotation is not obtained by chance. The genes coding for enzymes involved in phenol metabolism were selected from these two annotation approaches. Furthermore, the transcripts annotated by the methodologies mentioned above were aligned to the genome of the IHEM 14462 strain of *S. apiospermum* (genome accession number GCF_000732125.1) through Nucleotide Blast (28) to verify the identity of the transcripts with this genome annotation.

### Differential gene expression analysis

The differential expression (DE) among samples grown with phenol or glucose was quantified using EdgeR (29), part of the Bioconductor project. The DE value for each gene was calculated as the log_2_ of the fold change (log_2_FC). Thresholds of 1 and −1 of the log_2_FC were chosen to identify upregulated and downregulated genes respectively as previously described (30). The significance of the DE was assessed using the FDR (adjusted *P* value) statistic at the 5% level (30). Trimmed Mean of *M* values (TMM) was used in EdgeR for the normalization by library sizes among samples.

### Confirmation of differentially expressed genes

Real-time PCR (qPCR) was used to evaluate the DE profile of several genes of interest when the fungus was grown in the presence of phenol (experimental condition). The genes evaluated were those encoding for phenol 2-monooxygenase (XM_016790299.1), catechol 1,2-dioxygenase (XM_016788078.1), 3-oxoadipate enol-lactonase (XM_003712736.1), and hydroxyquinol 1,2-dioxygenase (XM_016791156.1). Primers were designed based on the annotated CDS from the clinical strain’s genome (Table S1). The *S. apiospermum* gene that encodes for actin (SAPIO_CDS5838 - XM_016788081.1) was used as a normalizer.

RT-qPCR assay was implemented as the growth assay for RNA-seq. The fungus was grown in MMS medium supplemented with either phenol (experimental condition) or glucose (control condition) with three replicates for each condition. When the fungus reached its exponential phase, the biomass was collected, and then RNA extraction was performed. The fungal mycelium collected was pulverized using liquid nitrogen. Then, the RNA was isolated using TRIzol following the manufacturer’s recommendations (Thermo Fisher Scientific). The methodology consisted mainly of adding TRIzol to the macerated tissue, followed by purification and precipitation of RNA with chloroform and isopropanol. After the extraction, quality and concentration were evaluated. The quality of the total RNA extracted was assessed by denaturing gel electrophoresis in an agarose gel with 0.5% of commercial bleach as proposed by Aranda et al. (31). The concentration of RNA was measured by absorbance at 260 and 280 nm through the Nanodrop Tm 2000. The RNA obtained from the TRIzol extraction was within an appropriate concentration range and complied with the quality standards required to proceed with the following methodologies.

The reverse transcription to cDNA was done using the RevertAid First Strand cDNA Synthesis kit using the oligo-dT primers (Thermo Fisher Scientific) following the manufacturer’s recommendations. A preliminary evaluation of the gene expression was done by PCR amplifying the genes of interest. The amplification of each of the genes was confirmed by electrophoresis in a 2% agarose gel.

The qPCR quantification was done using the CFX Opus 96 thermocycler (Bio-Rad). The amplification program used was as follows: an initial denaturation (step 1) at 94°C for 5 min; denaturation (step 2) at 94°C for 30 s; annealing/extension (step 3) at 58°C for 1 min; 39 cycles for steps 2 and 3. Melting curve: 65–95°C, 0.5°C increments for 5 s. The reactions were performed using Semi-Skirted 96-well PCR Plates with a final reaction volume of 10 µL. The amplification was done using the SsoAdvanced Universal SYBR Green Supermix (Bio-Rad). The standard curve consisted of five concentration points measured at 1,000, 500, 250, 125, and 62.5 ng/µL. Each gene of interest was quantified in an individual plate with the normalizer gene. Each of the samples was served by triplicate.

The analysis of the raw qPCR data were done using the CFX Maestro Software 2.3 for CFX Real-Time PCR Instruments (https://www.bio-rad.com/es-co/product/cfx-maestro-software-for-cfx-real-time-pcr-instruments?ID=OKZP7E15). The DE was calculated using the Pfaffl method (32), normalized by an internal control gene (actin). This method uses the efficiency calculated with the standard curve and the ΔΔCq (32). Normalized expression (ΔΔCq) represents the relative quantity of the target gene compared to the quantities of reference genes in the same biological system (33). The visualization of the DE between the experimental (phenol) and control (glucose) conditions was done with the R package ggplot2 (34).

### Quantification of phenol removal and identification of the degradation metabolite

To evaluate the production of hydroquinone as an intermediate metabolite during phenol degradation by HDO1, a phenol removal assay was performed. Phenol concentrations were quantified using the 4-aminoantipyrine (4-AAP) colorimetric method, while hydroquinone production was analyzed by liquid chromatography. HDO1 was initially grown on solid PDA for 20 days at 30°C. After this incubation period, the strain was transferred to two different experimental conditions: (i) fungal cultures in MMS medium supplemented with phenol at a final concentration of approximately 100 ppm (experimental condition), and (ii) MMS with phenol at 100 ppm without fungal inoculation (control) (Fig. S2). The 100 ppm phenol solution used in both conditions was prepared by diluting a 1,000 ppm phenol stock solution, mixing 10 mL of the stock with 90 mL of MMS. For the experimental condition, three agar plugs from the PDA culture were used to inoculate 100 mL of the MMS-phenol solution in 250 mL Erlenmeyer flasks. Each condition was performed in triplicate (Fig. S2).

From each sample, 2.3 mL was collected on days 0, 2, 3, 4, and 8 after the experiment was setup. On each sampling day, the samples were centrifuged at 7,000 rpm for 5 min and 30 s in 15 mL Falcon tubes. From the supernatant, 100 µL was used for phenol quantification, and the remaining 2.2 mL was stored at 4°C in the dark for subsequent analysis by chromatography. Only the samples collected on days 0 and 4 were used for chromatography.

The phenol quantification method using 4-aminoantipyrine was adapted from reference 35. The 100 µL of supernatant was diluted with 900 µL of distilled water (1:10) to obtain a final volume of 1 mL. To the diluted sample, 6 µL of 2% 4-aminoantipyrine was added, followed by 65 µL of 2 N ammonia, and mixed by vortex for 10 s. Then, 20 µL of 2% potassium ferricyanide was added to complete the reaction. The absorbance of the resulting mixture was measured at 510 nm using a P1 UV-Vis Spectrophotometer (MAPADA). Phenol concentration was determined using a calibration curve prepared within the range of 0–100 ppm. Statistical significance of the differences in phenol concentration between experimental and control samples was assessed using a Student’s *t*-test (*P* value < 0.05).

Hydroquinone production was monitored using HPLC (Agilent 1260, USA) with a Prontosil Eurobond C18 column (125 mm × 4.0 mm, 5 µm), from supernatants collected on days 0 and 4. 1.5 mL of each of the samples was filtered through 0.22 µm filters and loaded in the column. The mobile phase consisted of ultrapure water and methanol, with a volume ratio of 65/35 at the flow rate of 1 mL/min. The injection volume was 10 µL, and a diode array detector with a detection wavelength of 270–290 nm was used. The identification of phenol and hydroquinone was determined by the standard retention time method, and relative peak areas were used for phenol degradation.

## RESULTS

### *S. apiospermum* HDO1 growth

Although the fungus is capable of growing with phenol as sole carbon source, its growth was reduced compared to its growth with glucose as a carbon source (Fig. S3). This is because phenolic compounds act both as a carbon source for microorganisms and as microbicidal agents (36). The exponential growth phase of the HDO1 strain was found to start on the third day and continued up to the fifth in MMS supplemented with either glucose or phenol as the sole carbon source (Fig. S3). The exponential growth phase is characterized by increased biomass and the active metabolism of the carbon and energy sources, in this case, glucose or phenol. We decided to take samples for transcriptomics on the fourth day of growth since the fungus is in its active phase of metabolism.

### Transcriptome assembly and abundance

Between 61,901,286 and 85,879,374 reads were obtained from samples sequencing. After the quality trimming, between 43,447,630 and 62,484,106 reads remained per sample (Table S2). A total of 35,577 transcriptomic contigs were assembled from reads of both conditions for *S. apiospermum* HDO1. These assembled contigs had a 52.01% GC content and a median length of 12,691 nucleotides. The *N*_50_ for this data set was 23,622. From the contigs assembled, 212,384 ORFs were identified. Detailed information about the RSEM mapping percentage to the transcriptome assembly per sample is shown in Table S2.

### Functional annotation

**Fig 2:**
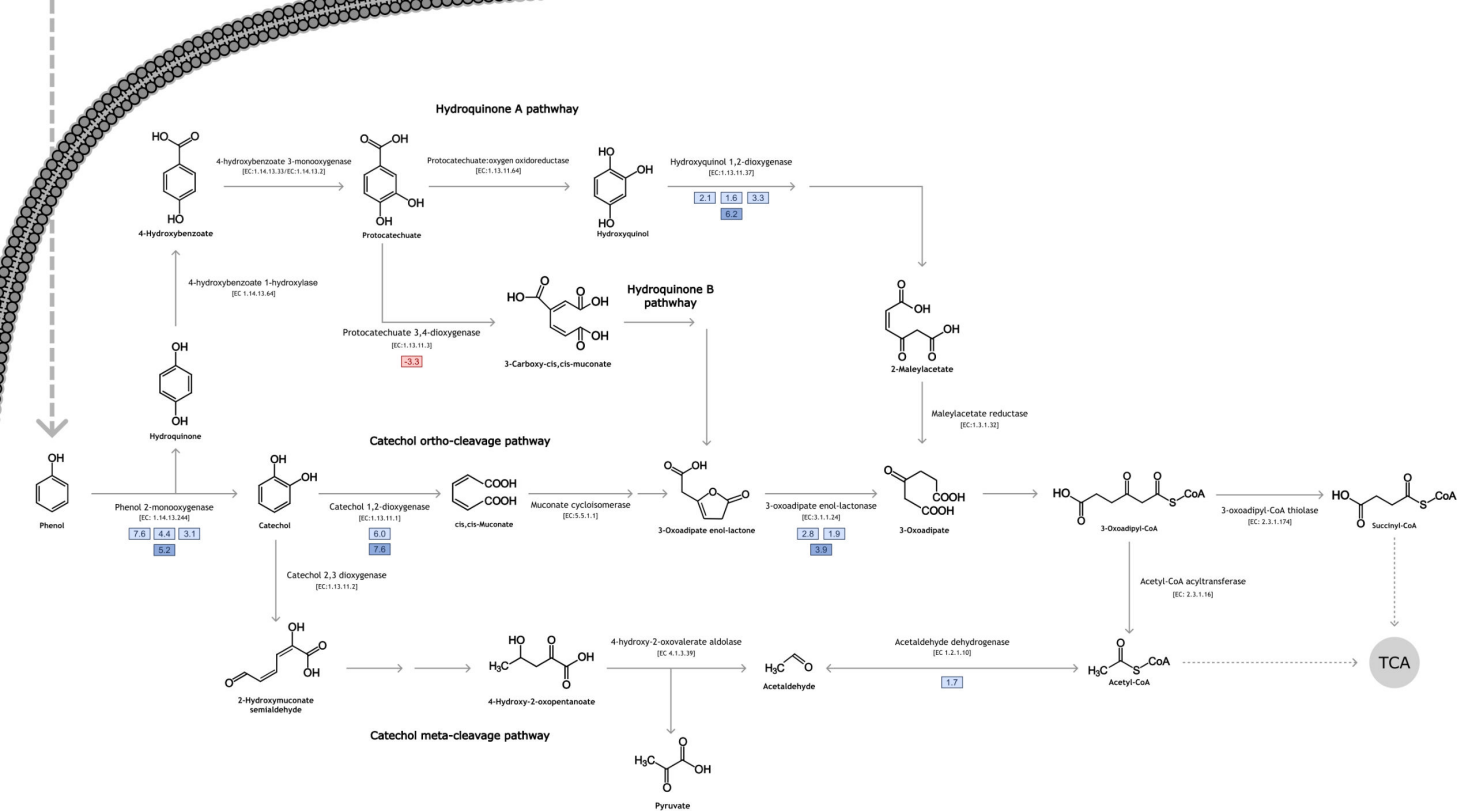
Phenol degradation pathways. Overexpressed genes are marked with light blue boxes (analyzed by RNA-seq) and dark blue boxes (analyzed by RT-qPCR), and the repressed gene is marked with a light red box. The log_2_FC values of the differentially expressed genes are indicated within each box. The dotted lines indicate the metabolites that can enter the tricarboxylic acid cycle (TCA).

**Fig 3:**
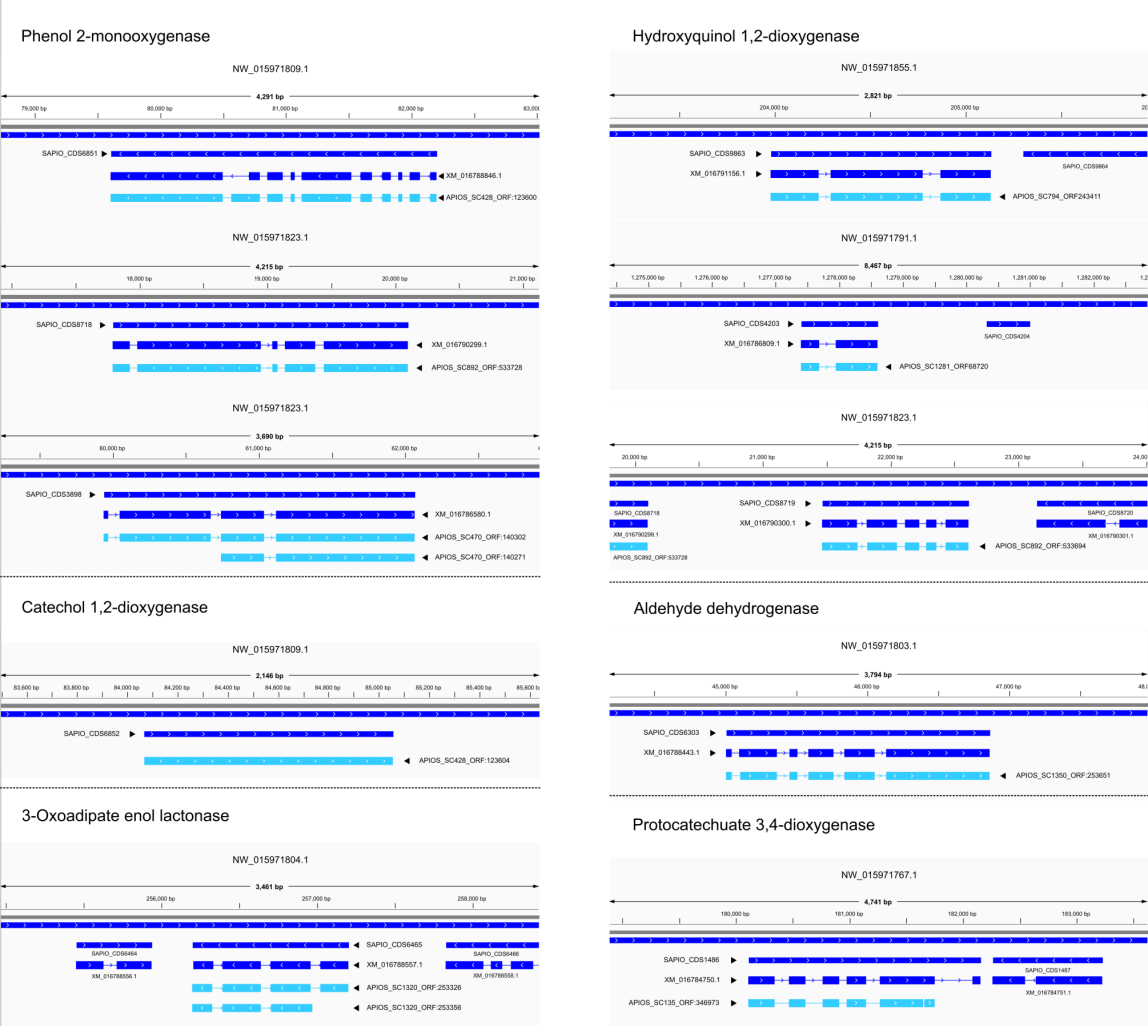
Visualization of the ORFs mapped to the clinical strain genome and annotated mRNAs. Genomic contigs of the clinical strain are named NW_* (blue). Annotated genes and mRNAs of the clinical strain genome are named SAPIO_CDS* and XM_*, respectively (blue). ORFs identified in the HDO1 environmental strain are named APIOS_SC* (cyan). Aligned regions are represented as boxes, and the gaps as thin lines. Arrows indicate the reading direction.

ORFs were functionally annotated using PANNZER2 and Eggnog (24, 25) (Table S3). Twenty-three transcripts coding for the enzymes involved in the phenol degradation (Table 1) were found among the identified ORFs. Six genes for the phenol 2-monooxygenase, the enzyme that begins the phenol degradation (Fig. 2), were annotated (Table 1). Of these, one of the genes has three isoforms (Table 1). These isoforms are APIOS_SC470_ORF:140302, APIOS_SC470_ORF:140271, and APIOS_SC470_ORF:140257, which share the same annotation and are mapped to the same mRNA (XM_016786580.1) annotated in the genome of the clinical strain (Table 1, gray colored cells). APIOS_SC470_ORF:140271 has the same length as XM_016786580.1 mRNA (1,908 pb) (Table 1, Fig. 3). Other ORF IDs sharing the same annotation are APIOS_SC861_ORF:520629, APIOS_SC861_ORF:521037, and APIOS_SC861_ORF:520585. These three transcripts mapped to phenol monooxygenase XM_016783331.1 on the clinical strain genome (Table 1; gray-colored cells). The APIOS_SC861_ORF:520629 has the same length (2,091 bp) as the XM_016783331.1. The last transcripts of phenol 2-monooxygenase that shared the same annotation are APIOS_SC585_ORF:574842 and APIOS_SC585_ORF:575030. Both mapped to XM_016786197.1, but APIOS_SC585_ORF:574842 has the same length (1,962 bp) as this mRNA (Table 1).

**TABLE 1:** ORFs annotated for enzymes involved in the phenol degradation pathway^*a*^

Pathway	Gene	ORF ID HDO1	Length	PANNZER and EggNOG annotation			BLAST against IHEM 14462 clinical genome					
				EggNOG 1 or/and PANNZER description	*E* value (EggNOG)	PPV (PANNZER)	mRNA ID of IHEM	Annotation in the genome of strain IHEM 14462	mRNA length	*E* value	Query cover	Identity
Catechol/hydroquinone	Gene encoding for phenol 2-monooxygenase	APIOS_SC428_ORF:123600	2,013	EggNOG and PANNZER: Phenol 2-monooxygenase (1.14.13.7)	1.2E−277	0.4620	XM_016788846.1 XP_016641483.1	Uncharacterized protein SAPIO_CDS6851 (PROVISIONAL). Conserved domain annotation: Phenol 2-monooxygenase; Phenol hydroxylase; Rossmann-fold NAD(P)(+)-binding proteins.	1,872	0	92%	99.08%
	Gene encoding for phenol 2-monooxygenase	APIOS_SC892_ORF:533728	2,043	EggNOG and PANNZER: Phenol 2-monooxygenase (1.14.13.7)	1.7E−298	0.5218	XM_016790299.1 XP_016640059.1	Phenol 2-monooxygenase (PROVISIONAL). Conserved domain annotation: Phenol 2-monooxygenase; Phenol hydroxylase; Rossmann-fold NAD(P)(+)-binding proteins.	2,043	0	100%	98.78%
	Gene encoding for phenol 2-monooxygenase	APIOS_SC470_ORF:140302	1,251	EggNOG and PANNZER: Phenol 2-monooxygenase - FAD binding domain-containing protein	3.2E−183	0.4812	XM_016786580.1 XP_016643556.1	FAD binding domain-containing protein (PROVISIONAL). Conserved domain annotation: Phenol 2-monooxygenase; Phenol hydroxylase; Rossmann-fold NAD(P)(+)-binding proteins.	1,908	0	100%	99.84%
		APIOS_SC470_ORF:140271	1,908	EggNOG and PANNZER: Phenol 2-monooxygenase - FAD binding domain-containing protein	5.8E−293	0.4789	XM_016786580.1 XP_016643556.1	FAD binding domain-containing protein (PROVISIONAL). Conserved domain annotation: Phenol 2-monooxygenase; Phenol hydroxylase; Rossmann-fold NAD(P)(+)-binding proteins.	1,908	0	100%	99.84%
		APIOS_SC470_ORF:140257	1,845	EggNOG and PANNZER: Phenol 2-monooxygenase - FAD binding domain-containing protein	3E−286	0.4999	XM_016786580.1 XP_016643556.1	FAD binding domain-containing protein (PROVISIONAL). Conserved domain annotation: Phenol 2-monooxygenase; Phenol hydroxylase; Rossmann-fold NAD(P)(+)-binding proteins.	1,908	0	100%	99.84%
	Gene encoding for phenol 2-monooxygenase	APIOS_SC861_ORF:520629	2,091	EggNOG and PANNZER: Phenol 2-monooxygenase (1.14.13.7)	0	0.5732	XM_016783331.1 XP_016646097.1	Phenol hydroxylase (PROVISIONAL). Conserved domain annotation: Phenol 2-monooxygenase; Phenol hydroxylase; Rossmann-fold NAD(P)(+)-binding proteins.	2,091	0	100%	100%
		APIOS_SC861_ORF:521037	1,839	EggNOG and PANNZER: Phenol 2-monooxygenase (1.14.13.7)	1.6E−295	0.5767	XM_016783331.1 XP_016646097.1	Phenol hydroxylase (PROVISIONAL). Conserved domain annotation: Phenol 2-monooxygenase; Phenol hydroxylase; Rossmann-fold NAD(P)(+)-binding proteins.	2,091	0	100%	100%
		APIOS_SC861_ORF:520585	1,335	EggNOG and PANNZER: Phenol 2-monooxygenase (1.14.13.7)	2.4E−213	0.5519	XM_016783331.1 XP_016646097.1	Phenol hydroxylase (PROVISIONAL). Conserved domain annotation: Phenol 2-monooxygenase; Phenol hydroxylase; Rossmann-fold NAD(P)(+)-binding proteins.	2,091	0	100%	100%
	Gene encoding for phenol 2-monooxygenase	APIOS_SC585_ORF:574842	1,962	EggNOG and PANNZER: Phenol 2-monooxygenase - FAD binding domain-containing protein	0	0.4552	XM_016786197.1 XP_016644203.1	FAD binding domain-containing protein (PROVISIONAL). Conserved domain annotation: Phenol 2-monooxygenase; Phenol hydroxylase; Rossmann-fold NAD(P)(+)-binding proteins.	1,962	0	100%	100%
		APIOS_SC585_ORF:575030	1,269	EggNOG and PANNZER**:** Phenol 2-monooxygenase - FAD binding domain-containing protein	3.1E−218	0.4612	XM_016786197.1 XP_016644203.1	FAD binding domain-containing protein (PROVISIONAL). Conserved domain annotation: Phenol 2-monooxygenase; Phenol hydroxylase; Rossmann-fold NAD(P)(+)-binding proteins.	1,962	0	100%	100%
	Gene encoding for phenol 2-monooxygenase	APIOS_SC31_ORF:447118	1,419	EggNOG and PANNZER: Related to phenol 2-monooxygenase	7.4E−200	0.6688	XM_016788445.1 XP_016641879.1	2,4-dichlorophenol 6-monooxygenase (PROVISIONAL). Conserved domain annotation: FAD binding domain; Rossmann-fold NAD(P)(+)-binding proteins.	1,794	0	100%	98.80%
Catechol ortho cleavage	Gene encoding for catechol 1,2-dioxygenase	APIOS_SC428_ORF:123604	993	EggNOG and PANNZER: Catechol 1,2-dioxygenase	1.2E−145	0.7462	SAPIO_CDS6852	Pseudogen		0	100%	99.70%
	Gene encoding for 3-oxo adipate enol-lactonase	APIOS_SC1320_ORF:253356	648	PANNZER: 3-oxoadipate enol-lactone hydrolase-like protein		0.4803	XM_016788557.1 XP_016641848.1	Alpha/beta hydrolase fold family protein (PROVISIONAL). Conserved domain annotation: Pimeloyl-ACP methyl ester carboxylesterase; Abhydrolase_5; Alpha/beta hydrolase family.	819	0	97%	100%
		APIOS_SC1320_ORF:253326	819	PANNZER: 3-oxoadipate enol-lactone hydrolase-like protein		0.5981	XM_016788557.1 XP_016641848.1	Alpha/beta hydrolase fold family protein (PROVISIONAL). Conserved domain annotation: Pimeloyl-ACP methyl ester carboxylesterase; Abhydrolase_5; Alpha/beta hydrolase family.	819	0	100%	100%
	Gene encoding for 3-oxo adipate enol-lactonase	APIOS_SC554_ORF:575598	1,041	PANNZER: Related to 3-oxoadipate enol-lactonase II		0.6460	SAPIO_CDS4979, SAPIO_CDS4980	Pseudogen		0	100%	97.79%
Hydroquinone A	Gene encoding for hydroxyquinol-1,2-dioxygenase	APIOS_SC892_ORF:533694	891	EggNOG and PANNZER: Related to hydroxyquinol-1,2-dioxygenase (1.13.11.1)	6E−115	0.6572	XM_016790300.1 XP_016640060.1	Uncharacterized protein SAPIO_CDS8719 (PROVISIONAL). Conserved domain annotation: Chlorocatechol 1,2-dioxygenase; Hydroxyquinol 1,2-dioxygenase (1,2-HQD).	891	0	100%	98.99%
	Gene encoding for hydroxyquinol-1,2-dioxygenase	APIOS_SC794_ORF:243411	1,005	EggNOG and PANNZER: Hydroxyquinol 1,2-dioxygenase	1.4E−157	0.5092	XM_016791156.1 XP_016639000.1	Catechol (PROVISIONAL). Conserved domain annotation: Chlorocatechol 1,2-dioxygenase; Hydroxyquinol 1,2-dioxygenase (1,2-HQD.)	1,005	0	100%	100%
	Gene encoding for hydroxyquinol-1,2-dioxygenase	APIOS_SC1281_ORF:68720	960	EggNOG and PANNZER: Related to hydroxyquinol-1,2-dioxygenase (1.13.11.1)	7.5E−164	0.6142	XM_016786809.1 XP_016643785.1	Uncharacterized protein SAPIO_CDS4203 (PROVISIONAL). Conserved domain annotation: Catechol 1,2-dioxygenase; Peptidase associated domain: C-terminal domain of M14 N/E carboxypeptidase.	960	0	100%	99.58%
	Gene encoding for maleylacetate reductase	APIOS_SC43_ORF:424362	1,074	EggNOG and PANNZER: Maleylacetate reductase	2E−157	0.5156	SAPIO_CDS2196	Pseudogen	1,074	0	100%	98.70%
Catechol metha cleavage	Gene encoding for aldehyde dehydrogenase	APIOS_SC836_ORF:539822	1,545	EggNOG and PANNZER: Aldehyde dehydrogenase (1.2.1.16,1.2.1.20,1.2.1.79)	1.8E−199	0.4317	XM_016784706.1 XP_016645822.1	Succinate-semialdehyde dehydrogenase (NAD(+)) (PROVISIONAL). Conserved domain annotation: Mitochondrial succinate-semialdehyde dehydrogenase and ALDH family members 5A1 and 5F1-like.	2,589	3.0E−15	26%	64.89%
	Gene encoding for aldehyde dehydrogenase	APIOS_SC232_ORF:62240	1,494	EggNOG and PANNZER: Aldehyde dehydrogenase (1.2.1.3,1.2.1.5)	2.7E−237	0.4336	XM_016788686.1 XP_016641630.1	Aldehyde dehydrogenase (PROVISIONAL). Conserved domain annotation: ALDH subfamily; aldehyde dehydrogenase family 2 member.	1,494	0	100%	99.73%
	Gene encoding for aldehyde dehydrogenase	APIOS_SC1350_ORF:253651	1,485	EggNOG and PANNZER: Aldedh domain-containing protein	3.2E−81	0.4443	XM_016788443.1 XP_016641877.1	**U**ncharacterized protein SAPIO_CDS6303 (PROVISIONAL). Conserved domain annotation: ALDH_AldA-AAD23400; Streptomyces aureofaciens putative aldehyde dehydrogenase AldA (AAD23400)-like.	1,494	0	100%	99.13%
Hydroquinone B	Gene encoding for protocatechuate 3,4-dioxygenase	APIOS_SC135_ORF:346973	1,173	EggNOG: Protocatechuate 3,4-dioxygenase activity. PANNZER: Intradiol ring-cleavage dioxygenase, core.	3.4E−134	0.7393	XM_016784750.1 XP_016645507.1	Uncharacterized protein SAPIO_CDS1486 (PROVISIONAL). Conserved domain annotation: Intradiol_dioxygenase_like; Intradiol dioxygenase supgroup.	1,257	0	99%	98.98%

^*a*^ The gray-shaded cells are isoforms belonging to the same gene.

Four ORFs encoding for enzymes that partake in catechol ortho-cleavage (Fig. 2) were annotated: one for catechol 1,2-dioxygenase (APIOS_SC428_ORF:123604) and three for 3-oxo adipate enol-lactonase (Fig. 2 and 3). Two of those annotated ORFs appear to be isoforms, APIOS_SC1320_ORF:253356 and APIOS_SC1320_ORF:253326, that mapped both to XM_016788557.1 (Table 1, Fig. 3). For the catechol meta-cleavage, three different ORFs that encoded for aldehyde dehydrogenase were annotated (APIOS_SC836_ORF:539822, APIOS_SC232_ORF:62240, and APIOS_SC1350_ORF:253651) (Table 1, Fig. 3). For the hydroquinone A pathway (Fig. 2), three ORFs annotated as hydroxyquinol 1,2-dioxygenase (APIOS_SC892_ORF:533694, APIOS_SC794_ORF:243411, and APIOS_SC1281_ORF:68720) (Table 1, Fig. 3) and one ORF annotated as maleylacetate reductase were identified (APIOS_SC43_ORF:424362). Finally, for the hydroquinone B pathway (Fig. 2), only one ORF APIOS_SC135_ORF:346973 annotated as protocatechuate 3,4-dioxygenase was identified (Table 1, Fig. 3).

### Differential gene expression analysis

From 212,384 ORFs identified, only 99,391 passed the EdgeR filter. This filter discards ORFs to which not enough reads are mapped to be able to calculate the DE value. The DE value was calculated for the ORFs that passed this filter (Table S4). The DE analysis of genes that encoded phenol degradation enzymes showed marked differences between the isolates using either glucose or phenol as the sole carbon source. Out of these 99,391 ORFs, 13,284 were significantly (FDR < 0.05) differentially expressed (log_2_FC above 1 or below −1).

From the 23 ORFs annotated related to phenol degradation, 12 had a significant DE value (FDR < 0.05). Out of these 12, 11 were differentially overexpressed (Table 2). The ORFs overexpressed encode for enzymes involved in the catechol ortho- and meta-cleavage and hydroquinone A pathways (Table 2). This result indicates that the phenol degradation occurs through both catechol and hydroquinone pathways (A pathway, Fig. 2). The remaining annotated ORF was repressed with phenol as its only carbon source (B pathway, Fig. 2); this ORF encodes for the protocatechuate 3,4-dioxygenase (Table 2). This result suggests that phenol degradation does not occur through the hydroquinone B path (Fig. 2).

**TABLE 2:** Differential expression values of annotated ORFs^*a*^

Enzyme	Pathway	RNA-seq			
		ORF ID HDO1	mRNA ID of IHEM	Expression values	
				LogFC	FDR
Phenol 2-monooxygenase	Catechol and hydroquinone	APIOS_SC428_ORF:123600	XM_016788846.1 XP_016641483.1	7.627949826	4.23605E−22
		APIOS_SC892_ORF:533728	XM_016790299.1 XP_016640059.1	4.435687774	1.97491E-11
		APIOS_SC470_ORF:140302	XM_016786580.1 XP_016643556.1	3.12826221	0.000229844
		APIOS_SC470_ORF:140271	XM_016786580.1 XP_016643556.1	2.166597923	0.000710565
Catechol 1,2-dioxygenase	Catechol ortho cleavage	APIOS_SC428_ORF:123604	SAPIO_CDS6852	6.01017091	2.89488E-15
3-oxoadipate enol-lactonase		APIOS_SC1320_ORF:253356	XM_016788557.1 XP_016641848.1	2.768303623	0.004222298
		APIOS_SC1320_ORF:253326	XM_016788557.1 XP_016641848.1	1.937805611	0.004903093
Hydroxyquinol 1,2-dioxygenase	Hydroquinone A pathway	APIOS_SC794_ORF:243411	XM_016791156.1 XP_016639000.1	2.093957292	0.000952238
		APIOS_SC1281_ORF:68720	XM_016786809.1 XP_016643785.1	1.575449489	0.007623764
		APIOS_SC892_ORF:533694	XM_016790300.1 XP_016640060.1	3.26975834	0.009517329
Aldehyde dehydrogenase	Catechol meta cleavage	APIOS_SC1350_ORF:253651	XM_016788443.1 XP_016641877.1	1.731032725	0.017737522
Protocatechuate 3,4-dioxygenase	Hydroquinone B pathway	APIOS_SC135_ORF:346973	XM_016784750.1 XP_016645507.1	−3.367881074	4.96768E-05

^*a*^ |Log2FC| > 1 and FDR < 0.05. Log2FC: Base 2 logarithm of the fold change.

Regarding the annotation and DE analysis of other enzymes belonging to the pollutant degradation by *S. apiospermum*, two ORFs were found. First, an ORF encoding for an extracellular β−1,3-exoglucanase (APIOS_SC189_c2_ORF:367497_i1) found significantly overexpressed (log_2_FC: 2.43 and FDR: 1.93 × 10^−4^). Second, an ORF encoding for an enzyme of the CYP53 protein subfamily in *S. apiospermum* (APIOS_SC907_c0_ORF:85028_i1) that was also significantly overexpressed (log_2_FC: 4.21 and FDR: 1.32 × 10^−7^). This last gene was found by doing a tBlastn against the CYP531D2 protein sequence of *Aspergillus nidulans* (XP_663269 from https://drnelson.uthsc.edu/p450seqs-dbs/ with an identity of 36% and a similarity of 53%). This alignment was performed with the default parameters of tBlastn (28). Both genes are involved in benzoate degradation. In addition to these ORFs, two other ORFs were also found, corresponding to genes encoding benzoate 4-monooxygenase. Both ORFs, APIOS_SC848_c1_ORF:528768 (log_2_FC: −0.91 and FDR: 0.307) and APIOS_SC848_c1_ORF:528667 (log_2_FC: 0.45 and FDR: 0.619) did not pass the log_2_FC threshold (−1 and 1) and were not statistically significant. In contrast to the differentially expressed ORFs encoding β−1,3-exoglucanase and the CYP53 protein subfamily, the latter ORFs did not show DE. However, we report them here because benzoate 4-monooxygenase is one of the enzymes in the benzoate degradation pathway that produces 4-hydroxybenzoate, which is then transformed into hydroquinone by the action of 4-hydroxybenzoate 1-hydroxylase. The 4-hydroxybenzoate 1-hydroxylase could not be annotated, but benzoate 4-monooxygenase was annotated in the HDO1 transcriptome. The lack of DE of the genes encoding benzoate 4-monooxygenase suggests that activation of the hydroquinone pathway occurs via the initial action of phenol-2-monooxygenase rather than benzoate 4-monooxygenase.

### Confirmation of the differential gene expression

The genes overexpressed in RNA-seq and quantified by qPCR are those encoding for enzymes phenol 2-monooxygenase XM_016790299.1, catechol 1,2-dioxygenase XM_016788078.1, 3-oxoadipate enol-lactonase XM_003712736.1, and hydroxyquinol 1,2-dioxygenase XM_016791156.1. In addition to these genes, an amplification by RT-PCR was attempted for the gene that encodes for catechol 2,3-dioxygenase. This enzyme was not annotated but is part of the catechol meta-cleavage pathway (Fig. 2). Even though the same cDNA sample was used to amplify the ORFs for the enzymes mentioned above, this gene cannot be amplified. For this reason, its expression was not quantified.

**Fig 4:**
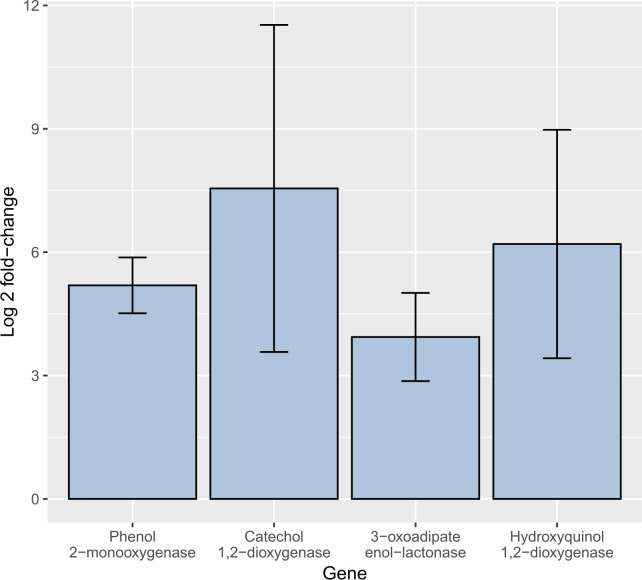
RT-qPCR quantification of selected differentially expressed genes in the experimental condition (phenol). The blue bars represent the log_2_ fold change of genes with phenol as the sole carbon source.

Regarding the expression quantification, the four genes evaluated were overexpressed in the fungal samples grown in medium with phenol as the only carbon source (Table 3, Fig. 4). These results corroborate the overexpression of genes that encode for phenol 2-monooxygenase, catechol 1,2-dioxygenase, 3-oxoadipate enol-lactonase, and hydroxyquinol 1,2-dioxygenase. This result supports that the phenol degradation occurs through the catechol ortho-cleavage and the hydroquinone A pathways.

**TABLE 3:** Differential expression values calculated from △△CT^*a*^

Enzyme	Pathway	RNA-seq				Quantification (RT-qPCR)			Expression in the presence of phenol
		ORF ID HDO1	mRNA ID of IHEM	Expression values		RT-PCR results	qPCR		
				LogFC	FDR		LogFC	*P* value	
Phenol 2-monooxygenase	Catechol and hydroquinone	APIOS_SC428_ORF:123600	XM_016788846.1 XP_016641483.1	7.627949826	4.24E−22	Specific amplification (XM_016790299.1)	5.19	0.007885	Overexpressed
		APIOS_SC892_ORF:533728	XM_016790299.1 XP_016640059.1	4.435687774	1.97E−11				
		APIOS_SC470_ORF:140302	XM_016786580.1 XP_016643556.1	3.12826221	0.000229844				
		APIOS_SC470_ORF:140271	XM_016786580.1 XP_016643556.1	2.166597923	0.000710565				
Catechol 1,2-dioxygenase	Catechol ortho cleavage	APIOS_SC428_ORF:123604	SAPIO_CDS6852	6.01017091	2.89E−15	Specific amplification (XM_016788078.1)^*b*^	7.55	0.001228	Overexpressed
3-oxoadipate enol-lactonase		APIOS_SC1320_ORF:253356	XM_016788557.1 XP_016641848.1	2.768303623	0.004222298	Specific amplification (XM_003712736.1— Pyricularia oryzae)^*b*^	3.94	0.000047	Overexpressed
		APIOS_SC1320_ORF:253326	XM_016788557.1 XP_016641848.1	1.937805611	0.004903093				
Hydroxyquinol 1,2-dioxygenase	Hydroquinone A pathway	APIOS_SC794_ORF:243411	XM_016791156.1 XP_016639000.1	2.093957292	0.000952238	Specific amplification (XM_016791156.1)	6.2	0.001465	Overexpressed
		APIOS_SC1281_ORF:68720	XM_016786809.1 XP_016643785.1	1.575449489	0.007623764				
		APIOS_SC892_ORF:533694	XM_016790300.1 XP_016640060.1	3.26975834	0.009517329				
Aldehyde dehydrogenase	Catechol meta cleavage	APIOS_SC1350_ORF:253651	XM_016788443.1 XP_016641877.1	1.731032725	0.017737522	Was not evaluated			Overexpressed
Protocatechuate 3,4-dioxygenase	Hydroquinone B pathway	APIOS_SC135_ORF:346973	XM_016784750.1 XP_016645507.1	−3.367881074	4.97E−05	Was not evaluated			Repressed

^*a*^ Fold change: amount of times the gene is expressed relative to the control condition (glucose). Log Fold-Change: Base 2 logarithm of the fold change.

^*b*^ A sequence from the clinical genome different from the one to which the RNAseq annotated sequence mapped. This sequence, however, shares the same annotation and corresponds to the same enzyme.

### Quantification of phenol removal and identification of degradation metabolite

Phenol was completely removed on the eighth day after the start of the experiment (*P* value: 0.000) (Fig. S4) as shown by the phenol removal assay in strain HDO1. Although phenol concentrations remained stable during the first 3 days, a significant decrease was observed on the fourth day (*P* value = 0.0172). According to chromatography, at day 0, both control and experimental samples exhibited a peak matching the retention time of the phenol standard (retention time: 4.45). On the fourth day of the assay, the control samples maintained the initial phenol concentration (Fig. S5). In contrast, the experimental samples still showed the phenol peak, but also a 34% decrease in phenol concentration compared to day 0 (Fig. S5). Furthermore, the HPLC results of the experimental samples also exhibited a new peak corresponding to hydroquinone (retention time: 1.468) (Fig. S6). These results confirm the production of hydroquinone as an intermediate in phenol metabolism.

## DISCUSSION

This study provides new insights into the strategies used by *S. apiospermum* to metabolize phenol when it is the sole carbon source. Through the DE analysis, it can be inferred that the phenol degradation by *S. apiospermum* HDO1 begins with the activity of the phenol 2-monooxygenase and follows through both the catechol-ortho ring cleavage and hydroquinone A pathways.

Considering the overexpression of the three genes that encode for phenol 2-monooxygenase, the enzyme catalyzing the conversion of phenol to catechol (the rate-limiting step in the phenol degradation pathway [37]), it can be inferred that the phenol degradation begins inside the cell since the phenol 2-monooxygenase is an intracellular enzyme (38). Although the fungus can secrete extracellular enzymes such as lignin peroxidase, manganese peroxidase, and laccases, the genes coding for these enzymes were not overexpressed with phenol as carbon source. These enzymes are known to participate in the transformation of polycyclic aromatic hydrocarbons (39). Another line of evidence supporting the intracellular phenol degradation is the overexpression of β−1,3-exoglucanase. The β−1,3-exoglucanase is involved in the fungal cell wall modifications through the hydrolysis of β-glucan (40, 41). Although phenol is a simple and hydrophobic molecule, its passing through the cell wall could induce changes in its structural conformation. This hypothesis requires additional experimental evidence.

Overexpression of genes coding for enzymes participating in the catechol ortho-cleavage and hydroquinone A pathway corroborates the findings of Claussen and Schmidt (8). They found by HPLC the production of *cis*,cis-muconic acid and 3-oxoadipate during the metabolization of phenol by *S. apiospermum*, leading them to propose that *S. apiospermum* metabolizes phenol simultaneously by the catechol ortho-cleavage and hydroquinone pathways (8). These results corroborate previous findings in other filamentous fungi, such as *Aspergillus fumigatus*, *Penicillium chrysogenum*, and *Penicillium simplicissimum*. Using chromatographic and enzymatic methods, it was found that various strains of these fungi degrade phenol through the catechol ortho-cleavage and the hydroquinone pathways (5, 42–44). At the time of Claussen and Schmidt's studies, it was not known that the catechol and hydroquinone pathways diverge and become ramified. Here we show that *S. apiospermum* metabolizes phenol through the catechol ortho-cleavage and the hydroquinone A pathway and not the hydroquinone B, as suggested by the silencing of the gene encoding for protocatechuate 3,4-dioxygenase. Interestingly, the phenol 2-monooxygenase might serve as a starting point for both the catechol and hydroquinone pathways. Although most reports on phenol 2-monooxygenase show the addition of oxygen in the ortho position forming catechol, some studies in bacteria have reported that phenol 2-monooxygenase catalyzes the conversion of phenol also in the para position simultaneously forming hydroquinone (45).

The overexpression of the gene encoding for the aldehyde dehydrogenase (log_2_FC: 1.73) can indicate that the route of the catechol meta-cleavage is also used by the fungus as part of the phenol degradation strategies. However, the aldehyde dehydrogenase enzyme is also involved in the metabolism of aromatic amino acids such as phenylalanine. According to the phenylalanine degradation pathway reported in KEGG (ec00360), one of the intermediate metabolites of this pathway is 4-hydroxy-2-oxopentanoate, which is also an intermediate of the catechol meta-cleavage pathway. This metabolite is converted into pyruvate and acetaldehyde (aldehyde) through the action of the 4-hydroxy-2-oxovalerate aldolase, which was not annotated in the transcriptome. The acetaldehyde produced by this reaction can be converted into acetyl-CoA by the action of the aldehyde dehydrogenase. Furthermore, the reaction catalyzed by the aldehyde dehydrogenase is reversible. For these reasons, the overexpression of the gene encoding for aldehyde dehydrogenase is not evidence of the activation of the catechol meta-cleavage pathway during phenol degradation. Although the lack of evidence for overexpression of the catechol 2,3-dioxygenase gene does not allow us to conclude whether the meta-catechol cleavage pathway is activated in the presence of phenol, the inability to amplify the gene by RT-PCR suggests that this route may not be present in strain HDO1. There are very few studies in fungi showing phenol metabolization from the meta-cleavage pathway. In fungi such as *Fusarium* sp. and *Graphium* sp., enzymatic activity of catechol 1,2-dioxygenase and catechol 2,3-dioxygenase has been found in mycelium grown on phenol, showing that catechol could be oxidized by ortho- and meta-fission (46, 47).

The overexpression of the gene encoding for the hydroxyquinol 1,2-dioxygenase APIOS_SC794_ORF:243411 indicates that phenol degradation occurs through the hydroquinone A pathway. These results agree with the findings by Claussen and Schmidt that *S. apiospermum* metabolizes phenol by hydroquinone pathways (8). However, overexpression of the gene of the CYP53 subfamily belonging to the P450 cytochrome (log_2_FC: 4.21) might suggest that the overexpression of hydroxyquinol 1,2-dioxygenase could be due to the degradation of benzoate. Hydroxyquinol 1,2-dioxygenase transforms hydroxyquinol into 3-hydroxy-cis,cis-muconate, and this compound is then converted into 2-maleylacetate. These reactions are shared by both pathways, the benzoate pathway and the hydroquinone A pathway. Although the components of the MMS medium do not contain benzoate, this compound is an intermediate in the degradation of aromatic compounds like phenylalanine, cinnamic acid (48), and toluene (49). According to the result of HPLC metabolite identification, the production of hydroquinone is evidence that the hydroquinone A pathway is active in the degradation of phenol by strain HDO1. It is not clear why the gene coding for the CYP53 subfamily is overexpressed. It is known that benzoate can be degraded through the CYP53 P450 subfamily as a response to intoxication in fungi (50). Also, it is known that the CYP53 gene is essential for the survival of fungal species (45). Considering this, the presence of phenol in fungal cells could activate the transcription of the CYP53 gene, because it is an aromatic compound similar to benzoate.

Despite the identification of the gene encoding hydroxyquinol 1,2-dioxygenase and the detection of hydroquinone as a metabolite in our study, most studies on filamentous fungi and yeasts conclude that phenol degradation occurs exclusively via the ortho-catechol cleavage pathway (1, 51, 52). Few studies report the hydroquinone pathway as one of the routes of phenol metabolization in microorganisms, and the few reports that exist are in fungi (5, 42–44). In addition to the overexpression of the gene encoding hydroxyquinol 1,2-dioxygenase and the detection of hydroquinone as a metabolite, the non-DE of the gene encoding benzoate 4-monooxygenase [EC:1.14.14.92] supports the hypothesis that phenol transformation starts with the production of catechol and hydroquinone by phenol-2-monooxygenase.

Hydroquinone can be formed either via phenol 2-monooxygenase, which converts phenol into hydroquinone, or via 4-hydroxybenzoate 1-hydroxylase, which transforms 4-hydroxybenzoate into hydroquinone (map00362). Although 4-hydroxybenzoate 1-hydroxylase could not be annotated, the gene of benzoate 4-monooxygenase—the enzyme responsible for converting benzoate into 4-hydroxybenzoate—was annotated and not differentially expressed in phenol degradation. This study provides important evidence that the hydroquinone pathway is among the metabolic routes activated during phenol degradation by *S. apiospermum*.

The phenol degradation pathway has been extensively studied in bacteria. Several studies in bacteria have reported the activation of catechol ortho-cleavage in phenol metabolism, while others describe bacterial phenol degradation via catechol meta-cleavage. Similar to our study, several bacterial species can also degrade phenol through two pathways simultaneously. For instance, in a consortium containing *Acidobacterium* sp. and *Chloroflexus* sp., degradation occurs via both ortho- and meta-cleavage (53). Another case of dual degradation pathways is observed in *Rhodococcus ruber*, where phenol from wastewater is degraded through both ortho- and meta-cleavage pathways (54). Another example of several degradation pathways is observed in *Pseudomonas stutzeri. P. stutzeri* degraded phenol rapidly through meta- and ortho-cleavage routes concurrently (55). Although bacteria and some fungi can metabolize phenol through two routes at the same time, few studies have found that bacteria can metabolize the contaminant through the hydroquinone route. Three previous studies addressed this topic. In the first, hydroquinone was detected as an intermediate metabolite of phenol degradation through CG-MS in *Acinetobacter pitti*. Although this metabolite was reported, the authors suggest that the metabolization route in *A. pitti* is thought to be meta-cleavage (56). In the second study, catechol and hydroquinone were identified as metabolites of phenol degradation in *Chromobacterium violaceum*, following the cloning and functional characterization of the phenol 2-monooxygenase gene. The authors concluded that phenol metabolism begins with either ortho- or para-hydroxylation, leading to the formation of catechol or hydroquinone, respectively (45). In the third, using a complementation experiment, a cluster of genes encoding monooxygenase proteins in specific strains of *Mycobacterium goodii* and *Mycobacterium smegmatis* was found to be involved in the conversion of phenol to hydroquinone through regioselective oxidation of phenol (57).

The finding of various genes (ORFs) encoding the same enzyme can be explained by gene duplication. This is not uncommon in both eukaryotes and prokaryotes (58). For example, in *Saccharomyces cerevisiae*, gene duplication is estimated to occur at a rate of 3.4 × 10^−6^ per gene/generation (58). These duplications are one of the main ways to increase the activity of inefficient enzymes (58). Such could be the case for the ORFs encoding for phenol 2-monooxygenases, for example.

The results of this study provide valuable information about the mechanisms involved in the tolerance and adaptation response of filamentous fungi to the presence of aromatic compounds such as phenol. Also, it represents an important advance in our understanding of the phenol degradation pathways utilized by *S. apiospermum*, providing knowledge for potential biotechnological applications focused on remediating phenol-contaminated environments.

## Data Availability

All data were uploaded to NCBI under BioProject number PRJNA1163296.

## SUPPLEMENTAL MATERIAL

Figures S1 to S6 aem.01038-25-s0001.docx Experimental designs, growth curve, phenol removal, and chromatograms.

Table S1 aem.01038-25-s0002.xlsx Primers for qPCR design.

Table S2 aem.01038-25-s0003.xlsx Number of reads before and after using Trimmomatic, SortmeRNA and RSEM.

Table S3 aem.01038-25-s0004.xlsx Complete PANNZER2 annotation.

Table S4 aem.01038-25-s0005.xlsx Differential gene expression values.

ASM does not own the copyrights to Supplemental Material that may be linked to, or accessed through, an article. The authors have granted ASM a non-exclusive, world-wide license to publish the Supplemental Material files. Please contact the corresponding author directly for reuse.
