# Supplementary material for: Deciphering the phenol degradation metabolic pathway in Scedosporium apiospermum HDO1
Source: Appl Environ Microbiol. 2025 Jul 10;91(8):e01038-25. doi: 10.1128/aem.01038-25 (PMC12366324; doi:10.1128/aem.01038-25)
Supplement: Figures S1 to S6 — Experimental designs, growth curve, phenol removal, and chromatograms. [file aem.01038-25-s0001.docx]

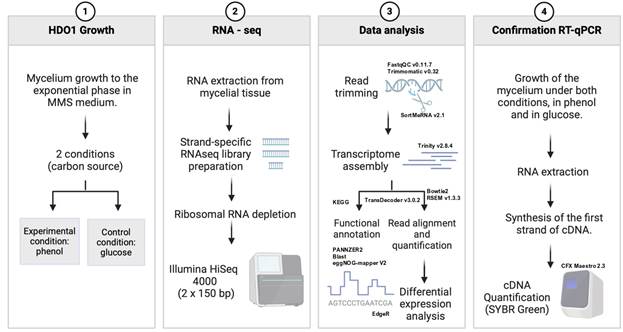


**Supplementary figure 1.** Experimental design and bioinformatic pipeline for the identification of phenol degradation pathways in *S. apiospermum*.


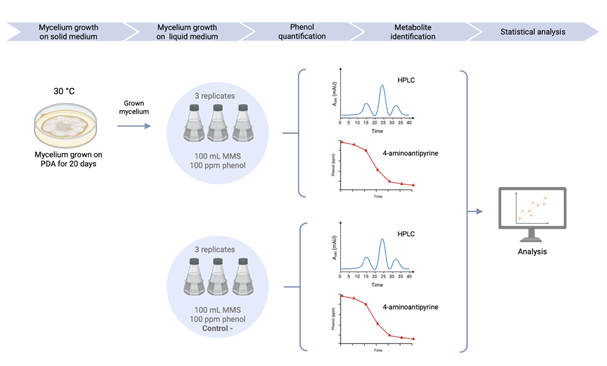


**Supplementary figure 2.** Experimental design for the phenol removal and metabolite identification in *Scedosporium apiospermum* HDO1. Two conditions were established, an experimental condition where the HDO1 strain was grown in MMS medium with 100 ppm phenol, and the negative control for the removal consisting of MMS medium with phenol at 100 ppm without the fungus. Samples were collected on days 0, 2, 3, 4 and 8 from the experiment set-up for phenol quantification by 4-aminoantipyrine. Furthermore, samples were collected on days 0 and 4 from the experimental set-up for metabolite identification using HPLC. Created in https://BioRender.com.


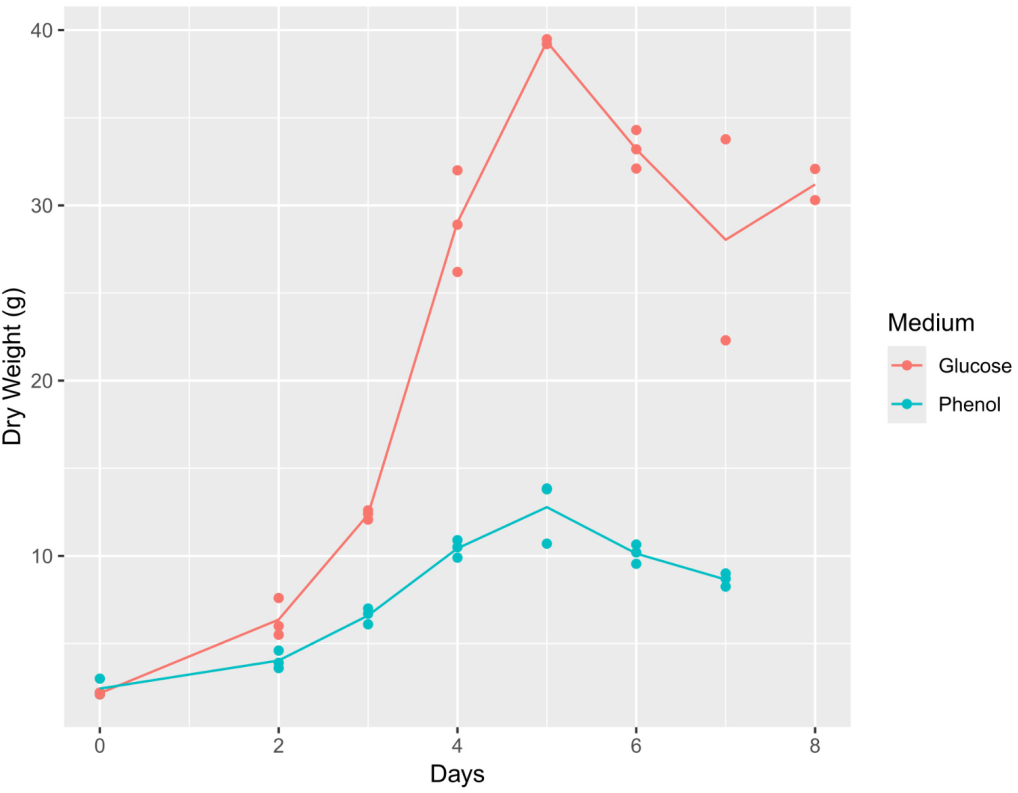
**Supplementary figure 3**. Growth curve of *S. apiospermum* in two conditions, phenol and glucose (control condition). The dry weight was determined in grams. Each point is the weight of each replicate.


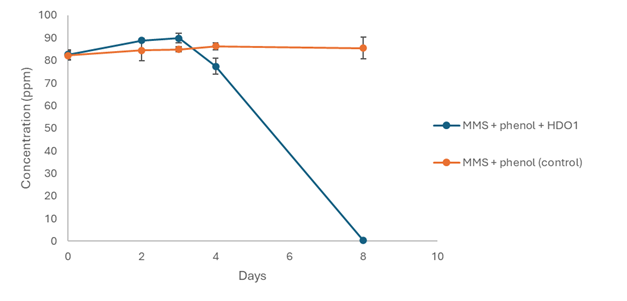
**Supplementary figure 4.** Phenol removal by *S.apiospermum* HDO1. The initial concentration was 100 ppm. Phenol quantification was measured after incubation for eight days and was compared in the presence and absence of mycelium. Points indicate the mean +- SD of three replicates from each condition (with and without HDO1 strain).


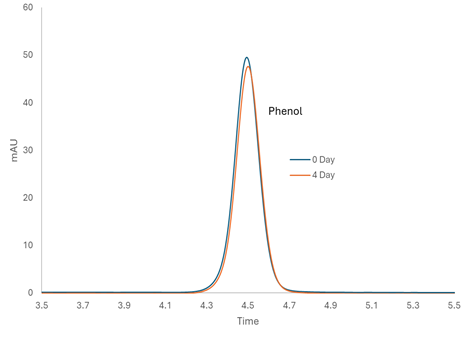


1. Control sample


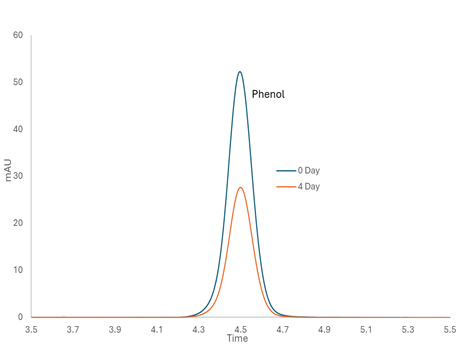


1. Experimental sample

**Supplementary figure 5.** Chromatogram of phenol concentration in both control and experimental samples. A. Control samples: there is no evidence of phenol degradation on the fourth day of the experiment (peak at 4.45 min) in the HPLC assay (Sample C1). B. Experimental samples: phenol degradation as shown by the reduction of its concentration (peak at 4.45 min) in the HPLC assay (Sample H1). Retention time in minutes (x-axis), and absorbance in milli-Arbitrary Units (y-axis).


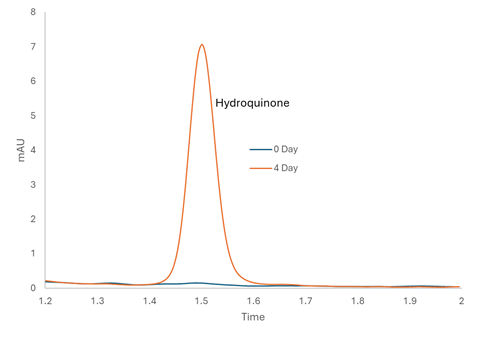


**Supplementary figure 6.** Chromatographic detection of hydroquinone as a phenol degradation product. Hydroquinone was detected on the fourth day of the assay (peak at 1.468 min) in experimental samples (MMS + phenol+HDO1). Retention time in minutes (x-axis), and absorbance in milli-Arbitrary Units (y-axis).
